# Therapeutic Potential of Kinkeliba (*Combretum micranthum* G. Don) Ethanolic Extract in Chronic DSS-Induced Colitis

**DOI:** 10.3390/molecules31091401

**Published:** 2026-04-23

**Authors:** Ibrahima Mamadou Sall, Meriem Aziez, Dragoş Hodor, Alina Diana Haşaş, Mara-Georgiana Haralambie, Semzenisi Ecaterina, Alexia-Teodora Hoța, Alexandru-Flaviu Tăbăran

**Affiliations:** 1 Department of Anatomic Pathology, Faculty of Veterinary Medicine, University of Agricultural Sciences and Veterinary Medicine of Cluj-Napoca, 400372 Cluj-Napoca, Roumania; dragos.hodor@usamvcluj.ro (D.H.); mara-georgiana.haralambie@usamvcluj.ro (M.-G.H.); ecaterina.semzenisi@student.usamvcluj.ro (S.E.); alexia-teodora.hota@student.usamvcluj.ro (A.-T.H.); 2 Laboratory of Plant Biotechnology and Ethnobotany, Faculty of Nature and Life Sciences, University of Bejaia, Bejaia 06000, Algeria; meriem.aziez@univ-bejaia.dz; 3 Department of Pathophysiology, Faculty of Veterinary Medicine, University of Agricultural Sciences and Veterinary Medicine of Cluj-Napoca, 400372 Cluj-Napoca, Romania; alina.hasas@usamvcluj.ro

**Keywords:** *Combretum micranthum* G. Don, ulcerative colitis, DSS-induced chronic colitis, CD3+ T-lymphocyte infiltration

## Abstract

**Background:** Kinkeliba (*Combretum micranthum* G. Don), commonly used in West African traditional pharmacopeia for its anti-inflammatory and gastrointestinal properties, remains poorly studied regarding its potential role in the prevention or treatment of ulcerative colitis. **Objective:** This study evaluated the therapeutic potential of the ethanolic extract of *Combretum micranthum* (EECM) in a murine model of chronic DSS-induced colitis. **Methods:** Male C57BL/6 mice were subjected to three cycles of 1.5% DSS administration over nine weeks to induce chronic colitis. EECM was administered orally at 50, 100, or 200 mg/kg during the final week. Disease severity was evaluated using the Disease Activity Index (DAI), colon length, biochemical and hematological markers, along with histopathological and immunohistochemical assessment of colonic tissue. **Results:** EECM treatment significantly improved clinical parameters and prevented colon shortening in chronic DSS-induced colitis. These improvements were associated with the restoration of serum biochemical and hematological profiles, along with reduced histopathological damage and preservation of colonic tissue architecture. Immunohistochemical analysis further demonstrated decreased CD3-positive T-lymphocyte infiltration in colonic tissue, suggesting modulation of local immune cell responses. **Conclusions:** These findings highlight the therapeutic potential EECM in ulcerative colitis and support further investigations to elucidate its mechanisms of action and evaluate its efficacy in future translational studies.

## 1. Introduction

Inflammatory bowel diseases (IBDs) are chronic relapsing inflammatory disorders of the gastrointestinal tract, characterized by alternating periods of inflammation and remission [1,2,3,4,5]. These conditions result from complex interactions involving genetic susceptibility, environmental influences, alterations in the intestinal microbiota, and dysregulated mucosal immune responses. These factors collectively drive sustained inflammation, epithelial barrier disruption, and progressive tissue injury, establishing a self-perpetuating cycle of intestinal damage [6,7,8]. Over time, this chronic inflammatory state alters the structural integrity of the intestinal mucosa and impairs its physiological functions, including nutrient absorption, barrier function, and immune regulation, thereby contributing to disease progression [9,10].

Ulcerative colitis (UC), a common form of IBD, is characterized by chronic inflammation and ulceration of the rectal and colonic mucosa, with continuous involvement of the colon. It clinically presents with abdominal pain, weight loss, hematochezia, and mucosal ulcerations [11,12,13]. The disease is characterized by a dysregulated immune response involving both innate and adaptive immune cells, leading to the overproduction of pro-inflammatory cytokines, including tumor necrosis factor-alpha (TNF-α), interleukin-1 beta (IL-1β), and interleukin-6 (IL-6) [14,15,16]. This inflammatory response is further exacerbated by oxidative stress, where reactive oxygen species (ROS) and reactive nitrogen species (RNS), produced during mitochondrial metabolism, drive epithelial injury and tissue damage. Intestinal dysbiosis, characterized by reduced bacterial diversity, promotes lipopolysaccharide (LPS) production, further enhancing ROS/RNS generation and cytokine release [17,18,19,20]. Concurrently, disruption of the intestinal epithelial barrier, mainly composed of tight junction proteins, facilitates the translocation of bacteria and antigens into the lamina propria, triggering further immune activation. Together, immune dysregulation, oxidative stress, microbial imbalance, and barrier disruption establish a vicious cycle that sustains mucosal damage and drives the chronic progressive nature of UC [21].

Current therapeutic strategies for UC, including aminosalicylates, corticosteroids, and immunomodulators, primarily aim to control inflammation and induce remission, but are often limited by incomplete efficacy, adverse effects, and long-term safety concerns [22,23]. In light of these challenges, there is a clear need for safer, more accessible, and sustainable therapeutic strategies. Consequently, naturally derived bioactive compounds have gained considerable attention for their potential to modulate immune pathways, reduce oxidative stress, and restore intestinal homeostasis. Recent evidence highlights the therapeutic potential of natural-derived compounds such as Hericium erinaceus, quercetin, berberine, and certain vitamins (e.g., biotin and niacin) in UC, due to their anti-inflammatory effects, modulation of the gut microbiota, and regulation of key inflammatory pathways [24]. In addition, these compounds generally exhibit improved tolerability and reduced systemic toxicity compared with conventional pharmacological treatments, making them promising candidates for novel interventions targeting the multifactorial nature of chronic intestinal inflammation [25,26,27].

*Combretum micranthum* G. Don, commonly known as kinkeliba, is a medicinal plant widely used in the traditional pharmacopeias of West Africa for its anti-inflammatory, digestive, diuretic, antidiabetic, and antihypertensive properties [28,29]. Phytochemical analyses have shown that its ethanolic extract is particularly rich in polyphenolic compounds, including hyperoside, quercitrin, ellagitannins, stilbenes, corilagin, sanguine H-4, and combretastatin B1, which are associated with antioxidant, anti-inflammatory, and immunomodulatory activities [30,31]. Experimental studies, including those from our group, have demonstrated that the ethanolic extract of *C. micranthum* (EECM) is safe, showing no significant acute or subacute oral toxicity, and can mitigate dextran sulfate sodium (DSS)-induced acute ulcerative colitis in C57BL/6 mice [32]. Furthermore, other investigations have reported that kinkeliba leaf extract alleviates skin inflammation both in vitro and in vivo, highlighting its anti-inflammatory potential, and also exhibits nephroprotective effects related to its antioxidant activity [31,33].

However, these studies remain limited to acute models or non-intestinal contexts, and the potential of *C. micranthum* in chronic intestinal inflammation remains largely unexplored. To date, its effects in DSS-induced chronic colitis have not been investigated using an integrated multi-parameter approach combining clinical, biochemical, hematological, histopathological, and immunohistochemical analyses.

Therefore, the present study was designed to evaluate the therapeutic efficacy of EECM in a DSS-induced chronic ulcerative colitis model in C57BL/6 mice. By addressing this gap, the study provides a comprehensive assessment of its effects in a chronic setting and contributes to a better characterization of its potential as a multi-target natural therapeutic agent.

## 2. Results

### 2.1. Established Phenolic Profile of EECM

The phenolic composition of the EECM was previously characterized in our earlier work [30] and is summarized in the Supplementary Materials Table S1. The extract was shown to possess a rich and diverse polyphenolic profile, predominantly composed of ellagitannins and stilbene derivatives, with hydroxybenzoic acids in lower proportions. This previously established phytochemical profile provides the scientific basis for evaluating its therapeutic potential in the present study.

### 2.2. Clinical and Macroscopic Evaluation of Chronic Colitis

**Figure 1:**
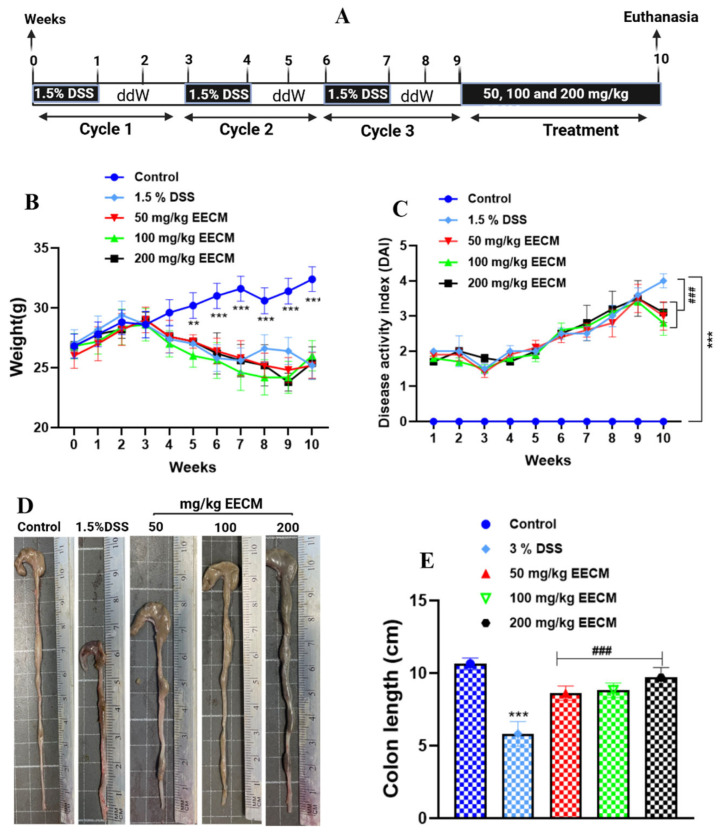
Effects of EECM on clinical and morphological parameters in DSS-induced chronic colitis. (**A**) Experimental design showing cyclic induction of chronic colitis with 1.5% DSS over nine weeks, followed by oral EECM treatment (50, 100, or 200 mg/kg) during the final week. (**B**) Body weight changes, (**C**) Disease Activity Index (DAI), (**D**) representative images of colon length, and (**E**) quantification of colon length. Data are expressed as mean ± SD (n = 5 per group). Statistical analysis was performed using one-way ANOVA followed by Dunnett’s multiple comparisons test. ### *p* < 0.001 vs. the DSS group; ** *p* < 0.01; *** *p* < 0.001 vs. the healthy control group.

Chronic colitis was induced by cyclic oral administration of 1.5% DSS over nine weeks, consisting of three cycles of one week of DSS exposure followed by two weeks of distilled water. The EECM (50, 100, or 200 mg/kg) was administered orally during the final week of the experiment prior to euthanasia (Figure 1A), allowing evaluation of its therapeutic effect on established disease. Body weight loss is a key clinical feature of chronic colitis and reflects the overall health condition of the animals. As shown in Figure 1B, all DSS-exposed groups exhibited progressive weight loss during the induction phase compared to the healthy control group (*p* < 0.001), confirming disease development. No differences were observed among DSS groups before treatment initiation. A slight temporary increase in body weight was observed in the untreated DSS group around weeks 8 and 9. Since all DSS groups were subjected to identical experimental conditions during that period, this fluctuation most likely reflects normal biological variation rather than real clinical improvement. During the treatment phase, mice receiving EECM showed stabilization and partial recovery of body weight (*p* < 0.001), whereas the untreated colitic group continued to lose weight. These findings indicate that EECM treatment limits body weight loss associated with chronic colitis.

Overall disease severity was further evaluated using the Disease Activity Index (DAI), which integrates body weight loss, stool consistency, and fecal blood. As presented in Figure 1C, DAI scores progressively increased in all DSS-treated groups throughout the induction phase compared to the healthy control group (*p* < 0.001), confirming successful establishment of chronic inflammation. After EECM administration during the final week, DAI values were significantly decreased in treated groups compared with the untreated DSS group (*p* < 0.001), indicating clinical improvement.

Colon shortening is a macroscopic indicator of sustained colonic inflammation and tissue damage. Representative images (Figure 1D) and quantitative measurements (Figure 1E) showed significant colon shortening in the untreated DSS group compared to the healthy control group (*p* < 0.001). EECM treatment significantly attenuated colon shortening in a dose-dependent manner (*p* < 0.001), demonstrating protection against inflammation-induced structural damage.

### 2.3. Biochemical Analyses

**Figure 2:**
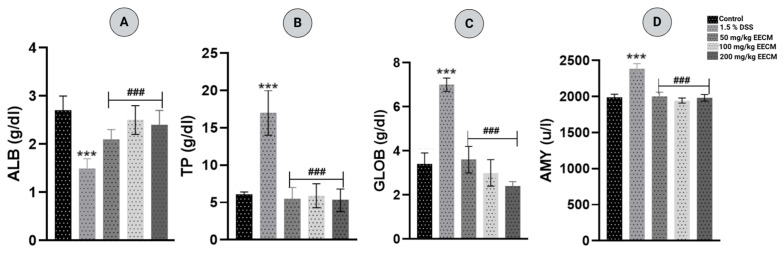
Effect of EECM on key biochemical parameters in DSS-induced chronic colitis. (**A**) Albumin (ALB), (**B**) Total Protein (TP), (**C**) Globulin (GLOB), and (**D**) Amylase (AMY) were measured to assess systemic biochemical alterations and the restorative effect of EECM. Data are expressed as mean ± SD (n = 5 per group). Statistical analysis was performed using one-way ANOVA followed by Dunnett’s multiple comparisons test. ### *p* < 0.001 vs. the DSS group; *** *p* < 0.001 vs. the healthy control group.

The biochemical profile analysis evaluated key biochemical markers to assess systemic alterations induced by DSS and the restorative effects of EECM treatment. Chronic colitis induced by 1.5% DSS caused significant disturbances in protein homeostasis and inflammatory markers. Specifically, serum albumin (ALB) levels were decreased (Figure 2A), while total protein (TP) (Figure 2B) and globulin (GLOB) levels (Figure 2C) were increased compared to the healthy control group (*p* < 0.001). These changes indicate systemic inflammation and impaired protein metabolism. Treatment with EECM at doses of 50, 100, and 200 mg/kg significantly mitigated these alterations (*p* < 0.001), partially restoring ALB, TP, and GLOB levels toward normal values. Similarly, the DSS-induced increase in serum amylase (AMY), reflecting inflammatory stress, was normalized by EECM treatment at all tested doses (*p* < 0.001), supporting its protective and anti-inflammatory potential.

### 2.4. Hematological Analyses

**Figure 3:**
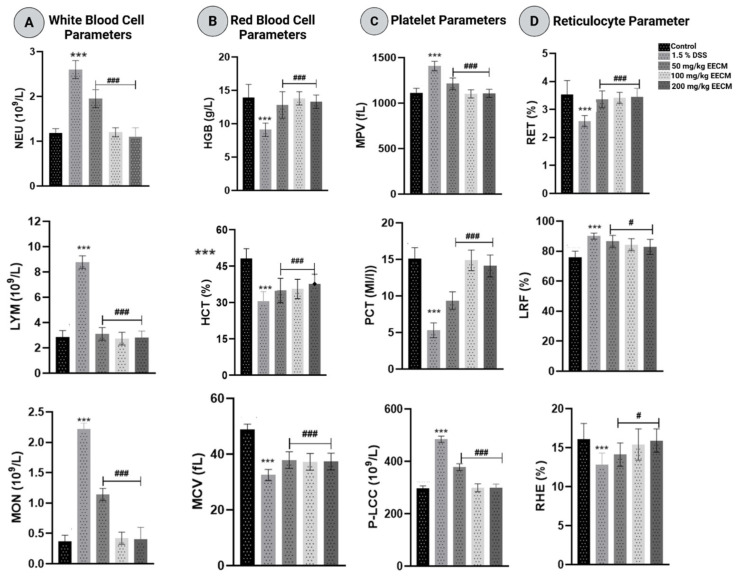
Effect of EECM on hematological parameters in DSS-induced chronic colitis. (**A**) White blood cell indices: neutrophils (NEUs), lymphocytes (LYMs), and monocytes (MONs). (**B**) Red blood cell parameters: hemoglobin (HGB), hematocrit (HCT), and mean corpuscular volume (MCV). (**C**) Platelet indices: mean platelet volume (MPV), platelet large cell count (P-LCC), and plateletcrit (PCT). (**D**) Reticulocyte parameters: reticulocyte percentage (RET), low fluorescence ratio (LRF), and reticulocyte hemoglobin equivalent (RHE). Data are expressed as mean ± SD (n = 5 per group). Statistical analysis was performed using one-way ANOVA followed by Dunnett’s multiple comparisons test. # *p* < 0.05; ### *p* < 0.001 vs. the DSS group; *** *p* < 0.001 vs. the healthy control group.

Induction of chronic colitis with 1.5% DSS resulted in significant hematological alterations consistent with a systemic inflammatory response, comparable to changes reported in patients with inflammatory bowel disease [34]. In white blood cell parameters, DSS exposure significantly increased neutrophil (NEU), lymphocyte (LYM), and monocyte (MON) counts (*p* < 0.001), reflecting activation of both innate and adaptive immune components. Administration of EECM at 50, 100, and 200 mg/kg (*p* < 0.001) significantly decreased these elevated leukocyte counts, indicating attenuation of DSS-induced systemic inflammation (Figure 3A).

Red blood cell indices were significantly affected by DSS treatment. Hemoglobin (HGB), hematocrit (HCT), and mean corpuscular volume (MCV) were significantly decreased (*p* < 0.001), suggesting inflammation-associated erythrocyte alterations compatible with anemia of chronic inflammation. EECM administration significantly improved these parameters (*p* < 0.001), restoring HGB and HCT levels and normalizing MCV values, thereby indicating protection against inflammation-induced erythrocyte dysfunction (Figure 3B).

Platelet-related parameters were also significantly altered following DSS exposure. The mean platelet volume (MPV) and platelet large cell count (P-LCC) were significantly increased (*p* < 0.001), indicating enhanced platelet activation and a pro-inflammatory state. In contrast, plateletcrit (PCT) was significantly reduced, reflecting altered platelet mass and turnover under inflammatory conditions. Treatment with EECM, particularly at 100 and 200 mg/kg (*p* < 0.001), significantly modulated MPV, PLCC, and PCT values, supporting its modulatory effect on inflammation-associated platelet dysregulation (Figure 3C).

Reticulocyte parameters were significantly altered following DSS exposure. Reticulocyte count (RET) and reticulocyte hemoglobin equivalent (RHE) were significantly decreased (*p* < 0.001), indicating impaired erythropoietic activity and reduced HGB synthesis under chronic inflammatory conditions. In contrast, Low Reticulocyte Fraction (LRF) was significantly increased, suggesting a shift in reticulocyte maturation dynamics. Treatment with EECM at 50, 100, and 200 mg/kg (*p* < 0.001) progressively restored RET and RHE levels while normalizing LRF values, supporting recovery of erythropoietic function and restoration of hematological homeostasis (Figure 3D).

### 2.5. Histological and Immunohistochemical Analyses

**Figure 4:**
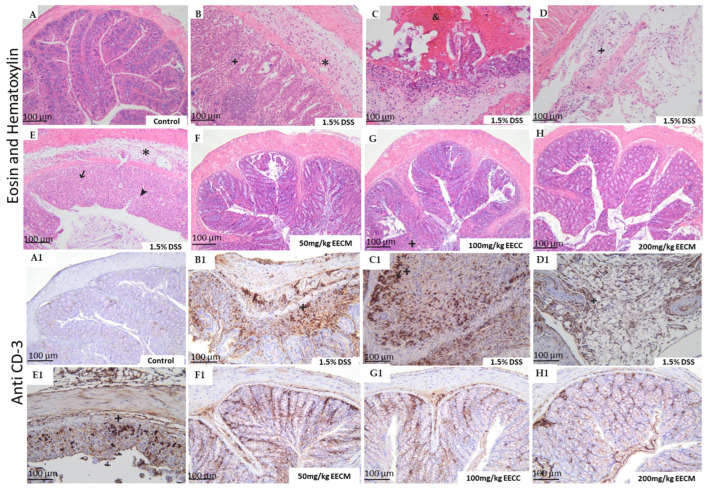
Histological and immunohistological analysis of colonic tissues in DSS-induced colitis and following treatment with EECM. Representative microphotographs of transverse colon sections stained with hematoxylin and eosin (H&E) staining and positive immunohistological staining as demonstrated by the anti-CD3 at different magnifications (G × 100). The control group shows normal colonic architecture without significant lesions. Symbols indicate ulceration (➔), hemorrhagic (&), inflammatory cell infiltration (+), edema (*), and crypt/goblet cell alterations (➤). Eosin and Hematoxylin: (**A**) Control; (**B**–**E**) 1.5% DSS; (**F**) 50 mg/kg EECM; (**G**) 100 mg/kg EECC; (**H**) 200 mg/kg EECM. Anti CD-3: (**A1**) Control; (**B1**–**E1**) 1.5% DSS; (**F1**) 50 mg/kg EECM; (**G1**) 100 mg/kg EECC; (**H1**) 200 mg/kg EECM.

Histological and immunohistochemical analyses of colon samples were performed to better characterize the impact of EECM at the tested doses (50, 100, and 200 mg/kg) on DSS-induced tissue lesions. The results shown in Figure 4, however, present the histological findings, hematoxylin-eosin (H&E) staining along with positive immunohistochemical labeling using anti-CD3 antibody.

In the healthy control group, no histological lesions were observed, indicating normal colonic architecture and intact tissue (Figure 4A). In contrast, the untreated colitic group (1.5% DSS) exhibited moderate structural damage characterized by extensive ulceration and marked inflammatory infiltration of the mucosal epithelium, lamina propria, and submucosa, composed of polymorphonuclear cells mixed with mononuclear leukocytes, as well as pronounced edema. Crypt dilation, crypt loss, and severe goblet cell depletion were also observed in a multifocal pattern (Figure 4B–E). Treatment with EECM extract at doses of 50, 100, and 200 mg/kg significantly improved the histological architecture of the mucosa, submucosa, and muscularis mucosae The treatment preserved crypt integrity, reduced submucosal edema, and attenuated inflammatory cell infiltration without causing architectural distortion (Figure 4F–H). These findings suggest that EECM exerts potential anti-inflammatory effects, likely attributable to its high phenolic content, thereby mitigating DSS-induced histopathological alterations and preserving the structural integrity of the colon.

Immunohistochemical staining for CD3 was performed to assess T-lymphocyte infiltration in colonic tissues. In the healthy control group, only a few CD3-positive cells were detected (Figure 4A1). In contrast, the DSS group showed increased CD3 immunoreactivity, with CD3-positive cells observed in the mucosal epithelium and lamina propria (Figure 4B1–E1). These cells correspond to T lymphocytes (LYMs) residing in the colonic tissue and involved in the inflammatory response associated with DSS-induced experimental colitis. In this model, T-cell infiltration is predominantly associated with pro-inflammatory responses, particularly involving Th1 and Th17 subsets, which contribute to mucosal damage and disease progression.

In the EECM-treated groups (50, 100, and 200 mg/kg), CD3-positive cells were also detected since these groups received DSS; however, EECM treatment reduced CD3 immunoreactivity and the infiltration of CD3-positive cells compared with the untreated DSS group (Figure 4F1,G1,H1).

**Figure 5:**
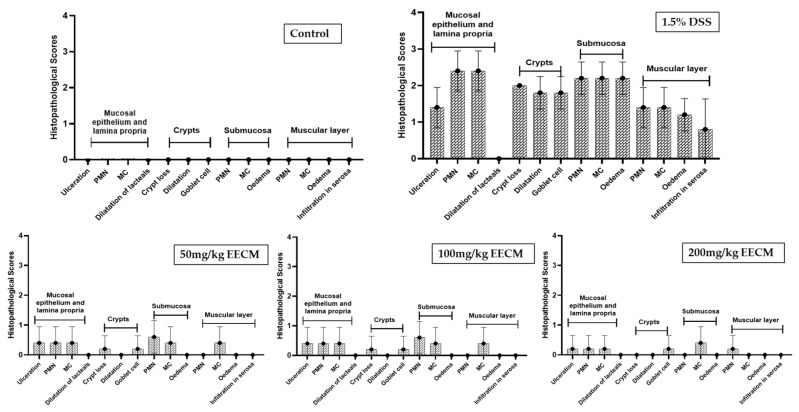
Histopathological scoring of colonic tissue in DSS-induced colitis and evaluation of the effect of EECM treatment. Histopathological alterations were graded according to severity as follows: 0 = no significant change, 1 = minimal, 2 = mild, 3 = moderate, and 4 = severe.

The quantification of histopathological scores provides quantitative support for the morphological alterations described above (Figure 5). The untreated DSS group showed a significant increase in histopathological scores compared to the control group (*p* < 0.001), confirming the severity of tissue damage. In contrast, treatment with EECM (50, 100, and 200 mg/kg) resulted in a significant and dose-dependent reduction in histopathological scores compared to the DSS group (*p* < 0.001). These quantitative findings corroborate the histological observations, indicating an overall attenuation of tissue damage in EECM-treated groups.

## 3. Discussion

This study evaluated the protective effects of EECM against chronic colonic inflammation using a well-established murine model of DSS-induced colitis. The cyclic administration of DSS over nine weeks was designed to mimic the relapsing course of human ulcerative colitis, enabling the assessment of both persistent tissue injury and systemic inflammatory responses [35,36]. EECM was administered during the final week to assess its therapeutic potential in the context of established colonic injury.

The cyclic administration of 1.5% DSS over nine weeks is considered to establish a robust model of chronic colitis that closely mimics the relapsing–remitting nature of human ulcerative colitis. Repeated cycles of DSS exposure induce recurrent epithelial injury, disrupting the mucosal barrier and increasing intestinal permeability, which allows luminal antigens and microbiota to penetrate the lamina propria and amplify local immune responses. Between cycles, recovery periods with distilled water permit partial mucosal healing, reflecting the dynamic interplay between tissue damage and spontaneous remission that characterizes chronic disease [34,37,38]. Over successive cycles, persistent immune activation, oxidative stress, and epithelial dysfunction lead to cumulative structural alterations, including crypt distortion, goblet cell depletion, submucosal edema, and progressive remodeling of the colonic wall [35,39]. This progressive accumulation of injury underpins both the clinical manifestations, such as weight loss, diarrhea, and fecal blood, and the systemic inflammatory and hematological disturbances observed in the model [40]. The DSS-induced chronic colitis therefore represents a relevant experimental platform to investigate interventions targeting epithelial integrity, immune modulation, and oxidative stress in the context of sustained intestinal inflammation.

Building on the previously established chemical and functional profile of EECM, its rich content of polyphenols, including ellagitannins, stilbene derivatives, and hydroxybenzoic acids, may provide a mechanistic basis for the extract’s in vivo activity [32]. These bioactive compounds are recognized for their potential to modulate oxidative stress, preserve epithelial barrier integrity, and regulate local inflammatory responses, making them particularly relevant in the context of chronic DSS-induced colitis [18,41,42]. In this model, the DAI serves as a key parameter for assessing disease severity, reflecting intestinal dysfunction caused by mucosal injury. DSS is known to disrupt the epithelial lining, increasing gut permeability and potentially allowing luminal antigens and bacteria to trigger local inflammation. This manifests as diarrhea due to impaired absorption and fluid secretion, while mucosal injury and microvascular disruption cause the presence of blood in the stool. Weight loss arises from reduced nutrient uptake and the systemic consequences of chronic inflammation [43,44]. In line with our previous observations in acute colitis, treatment with EECM significantly reduced the DAI, suggesting an alleviation of intestinal damage and a partial restoration of barrier function [30]. These effects are consistent with the proposed role of EECM’s phenolic compounds in preserving epithelial integrity and mitigating inflammatory responses in DSS models.

Consistent with the clinical improvement reflected by the DAI, colon length provides a macroscopic measure of the severity of intestinal inflammation. Repeated DSS exposure induces persistent mucosal injury and inflammatory infiltration, promoting tissue edema, muscular contraction, and progressive remodeling of the colonic wall, which results in shortening. This structural alteration is widely used as a marker of cumulative inflammatory damage in experimental UC and aligns with previous studies showing that colon shortening correlates closely with the extent of mucosal inflammation and tissue injury [43,45]. The attenuation of colon shortening following EECM treatment suggests that the extract may reduce inflammation-associated structural changes. Similar protective effects on colon morphology have been reported in the DSS acute model, where preservation of epithelial integrity and reduction in mucosal infiltration maintained normal colon architecture [30]. Such effects may be attributed to the phenolic constituents of EECM, particularly ellagitannins and stilbene derivatives, which have been shown in experimental colitis to reduce epithelial damage and limit inflammatory tissue remodeling, thereby preserving colon structure [42,46,47]. Collectively, these findings demonstrate that EECM may exert a protective effect against chronic inflammatory damage in the colon while supporting epithelial and tissue homeostasis.

The biochemical alterations observed in DSS-induced chronic colitis reflect the systemic impact of intestinal inflammation beyond the colon. Hypoalbuminemia is commonly reported in experimental and clinical forms of UC and is generally associated with impaired protein synthesis, increased intestinal permeability, and intestinal loss of plasma proteins due to mucosal damage [48,49]. At the same time, the increase in TP and GLOB levels observed after DSS exposure may reflect the enhanced production of acute-phase and immune-related proteins during the inflammatory response. These alterations suggest a disruption of normal protein homeostasis driven by sustained immune activation. Similar changes have been reported in DSS-induced colitis as well as in patients with inflammatory bowel disease, where decreased ALB and elevated GLOB fractions serve as indicators of ongoing inflammation [49,50,51].

Similarly, the elevation of AMY in DSS-treated animals may reflect systemic inflammatory stress and altered digestive enzyme regulation, occasionally reported in experimental colitis due to the close physiological interplay between intestinal inflammation and pancreatic function [52,53]. Treatment with EECM attenuated these biochemical disturbances, partially restoring ALB, TP, and GLOB levels, and normalizing serum AMY. These effects may be related to the polyphenolic constituents of EECM, which have been reported to improve systemic biochemical alterations in experimental colitis, including the modulation of circulating inflammatory mediators such as alanine transaminase (ALT), alkaline phosphatase (ALP), Aspartate Aminotransferase (AST), and Gamma-Glutamyl Transferase (GGT), and the restoration of intestinal barrier integrity [54]. Collectively, these findings suggest that EECM may contribute to the maintenance of systemic biochemical homeostasis during chronic intestinal inflammation, in agreement with our previous observations in acute colitis [30].

Chronic colitis induced by cyclic administration of 1.5% DSS over nine weeks resulted in significant hematological alterations, reflecting the systemic consequences of sustained intestinal inflammation. Elevated neutrophil, lymphocyte, and monocyte counts indicate persistent activation of both innate and adaptive immune responses, consistent with the recruitment of immune cells to the inflamed mucosa and systemic circulation observed in experimental and clinical IBD [55,56,57]. DSS-induced reductions in HGB, HCT, and MCV indicate inflammation-associated erythropoietic suppression, characteristic of anemia of chronic disease. This condition is driven by inflammatory cytokines, such as IL-6 and TNF-α, which are known to contribute to iron metabolism and inhibit erythroid progenitor differentiation [58,59]. Concurrent alterations in platelet indices, including increased MPV and PLCC, along with decreased plateletcrit PCT, reflect a pro-inflammatory and pro-thrombotic state. In parallel, reticulocyte dysregulation indicates impaired erythropoietic activity under chronic inflammatory stress, consistent with inflammation-associated anemia observed in experimental colitis [60,61]. Overall, these hematological changes reflect the systemic impact of prolonged epithelial damage, mucosal immune activation, and chronic cytokine-driven inflammation in the DSS model.

Administration of EECM during the final week reduced these systemic alterations, indicating a hematoprotective effect. Normalization of leukocyte counts reflects suppression of chronic inflammatory signaling, which may be associated with reduced pro-inflammatory cytokine production and preservation of mucosal immune balance. Recovery of red blood cell indices and reticulocyte counts suggests restoration of erythropoiesis, potentially related to decreased inflammatory inhibition of bone marrow progenitors and improved intestinal barrier integrity, which limits systemic immune activation. Modulation of platelet parameters further indicates decreased platelet activation and turnover, consistent with attenuation of chronic inflammation and pro-thrombotic alterations. These effects are consistent with previous reports on the anti-inflammatory activity of EECM extract [31,33], and align with our prior findings in acute colitis, where EECM improved hematological homeostasis alongside clinical and biochemical recovery [30].

Histological examination of the colon provides a direct measure of mucosal and submucosal integrity, offering critical insight into the extent of tissue damage induced by chronic DSS exposure. In the untreated DSS group, severe epithelial ulceration, extensive inflammatory infiltration of the mucosa, lamina propria, and submucosa, along with pronounced edema, crypt loss, and goblet cell depletion reflect the cumulative consequences of repeated cycles of epithelial injury and immune activation. These alterations are consistent with chronic inflammatory processes described in DSS models, where sustained epithelial disruption facilitates translocation of luminal antigens and bacteria, thereby amplifying local inflammation and promoting structural remodeling of the colonic wall [34,37,62,63]. Such histopathological patterns are also frequently reported in human ulcerative colitis, emphasizing the translational relevance of this model [38,43]. The presence of polymorphonuclear and mononuclear infiltrates, together with multifocal crypt architectural distortion, indicates ongoing activation of innate and adaptive immune components, mechanisms that have been widely documented in other chronic DSS studies.

Treatment with EECM at 50, 100, and 200 mg/kg significantly mitigated these histopathological alterations in a dose-dependent manner. Preservation of crypt architecture, reduction in submucosal edema, and attenuation of inflammatory cell infiltration suggest that the extract confers protection to colonic tissue against epithelial injury and limits mucosal immune activation. These effects are possibly related to the high content of polyphenols in EECM, particularly resveratrol, ellagic acid and *p*-hydroxybenzoic acid which have been reported in chronic colitis models to reduce oxidative stress, modulate pro-inflammatory cytokine expression, and stabilize epithelial tight junctions. Similar protective effects have been observed with polyphenol-rich extracts in DSS-induced chronic colitis, where reductions in mucosal inflammatory infiltrates and maintenance of crypt structure correlate with improvements in clinical and biochemical parameters [46,64,65]. The dose-dependent improvement of histopathological scores in EECM-treated groups further supports its potential role in mitigating chronic intestinal inflammation, preserving mucosal architecture, and maintaining colonic structural integrity, in line with our previous observations in acute colitis models [30].

Immunohistochemical staining for CD3 was performed to evaluate T-lymphocyte involvement in DSS-induced chronic colitis. In the untreated DSS group, intense CD3 immunoreactivity was detected throughout the mucosa, lamina propria, and submucosa, indicating substantial T-cell infiltration within the inflamed colonic tissue. This accumulation of CD3-positive LYMs reflects persistent activation of adaptive immune responses, a characteristic feature of chronic DSS-induced inflammation, where T cells contribute to the maintenance and amplification of the inflammatory cascade through cytokine production [64,65].

Administration of EECM reduced CD3-positive cell infiltration in a dose-dependent manner, suggesting that the extract attenuates T-cell-mediated immune activation within the colonic mucosa. This immunomodulatory effect may be related to the polyphenolic constituents of EECM, including resveratrol and ellagic acid, which have been shown to regulate inflammatory signaling pathways such as NF-κB and to potentially suppress T-lymphocyte recruitment in experimental colitis [44,66,67]. The reduction in T-cell infiltration observed in treated groups further supports a protective effect of EECM on mucosal immune homeostasis and is consistent with the histological improvements observed in DSS-induced colitis.

Taken together, these findings demonstrate the protective effects of EECM in chronic DSS-induced colitis, integrating clinical, macroscopic, biochemical, hematological, and histological observations. The results suggest that EECM exerts multifactorial protective effects by modulating intestinal inflammation and preserving colonic structural integrity. Future investigations will aim to fractionate the crude extract to identify its active constituents and subsequently evaluate their therapeutic efficacy in comparison with a positive pharmacological control, thereby allowing a rigorous comparison with standard pharmacological treatments.

## 4. Materials and Methods

### 4.1. Preparation of Ethanolic Extract from Combretum micranthum G. Don Leaves

The EECM leaves were prepared as previously described [28,29]. Briefly, powdered dried leaves were extracted with 70% (*v*/*v*) ethanol at a plant-to-solvent ratio of 1:40 (*w*/*v*) under continuous stirring for 2 h at room temperature. After filtration through Whatman filter paper, the extract was centrifuged at 3000× *g* for 10 min. The supernatant was subsequently concentrated at 50 °C using a rotary evaporator, and the resulting dry extract was stored at 4 °C until analysis.

### 4.2. Animals and Housing Conditions

Male C57BL/6 mice (6–9 weeks; 21–25 g) were obtained from the Institutul Național de Cercetare-Dezvoltare în Domeniul Patologiei și Științelor Biomedicale “Victor Babeș”. Animals were maintained in plastic cages under standardized environmental conditions, including a temperature of 23–25 °C, relative humidity of 55 ± 10%, and 12 h light/dark cycle with free access to food and water. Prior to the experimental procedures, mice were allowed to acclimatize for 14 days.

### 4.3. Experimental Design of DSS-Induced Chronic Colitis and Treatment

The mice were randomly divided into five experimental groups (n = 5 per group) using a simple randomization method (random allocation ensuring equal probability for each animal): a control group, a 1.5% DSS-treated group, and three groups receiving DSS reagent (molar mass 40,000 g/mol; Carl ROTH, Karlsruhe, Germany) and treated with the EECM at doses of 50, 100, and 200 mg/kg. The control group (non-colitic) received distilled water throughout the experiment. Chronic colitis was induced by oral administration of DSS at a concentration of 1.5% over a total period of nine weeks, following a cyclic protocol comprising three successive cycles. Each cycle consisted of one week of DSS administration (induction phase) followed by two weeks of distilled water (remission phase). EECM treatment was administered orally to the respective treatment groups during the final week of the experiment (week 10), after completion of the DSS induction phase, at doses of 50, 100, or 200 mg/kg for seven consecutive days.

After treatment, the animals were anesthetized by inhalation of a high dose of isoflurane in a sealed induction chamber. Adequate anesthesia was confirmed by the absence of pedal and corneal reflexes, after which cervical dislocation was performed to ensure euthanasia. All procedures were carried out in accordance with Directive 2010/63/EU of the European Parliament and of the Council (22 September 2010) on the protection of animals used for scientific purposes and complied with established ethical guidelines [68]. Blood samples were collected into EDTA-containing tubes for hematological analysis and into heparinized tubes for biochemical assays. The colon was excised and fixed in 10% neutral buffered formalin for histological examination [69].

### 4.4. Disease Activity Scoring

During the experimental period, the DAI was assessed to evaluate the severity of colitis through the integration of multiple clinical parameters, including body weight changes, stool consistency, rectal bleeding, piloerection, and reduced locomotor activity. These parameters were recorded daily for each animal. These parameters were scored according to previously established criteria and used to calculate the DAI [70].

DAI was calculated using scores for body weight loss (0 = none; 1= 1–5%; 2 = 5–10%; 3 = 10–15%; 4 = >15%), stool consistency (0 = normal; 1 = slightly loose stools; 2 = loose stools; 3 = very loose stools; 4 = watery diarrhea), and the presence of blood in stools (0 = none; 1 = slightly bloody; 2 = bloody; 3 = gross bleeding; 4 = massive bleeding in the stool) (Table 1), following the scoring system described by Zhou Y et al. [37]. Briefly, DAI = [(weight loss score) + (stool consistency score) + (blood score)]/3.

### 4.5. Biochemical and Hematological Assessment

Biochemical parameters were measured using an automated chemistry analyzer (Scil -Element RC, Viernheim, Germany), including ALB, TP, GLOB, and AMY.

Hematological parameters were assessed using an automated hematology analyzer (Diatron Abacus Junior 5, Budapest, Hungary), including white blood cell parameters (NEU, LYM, MON), red blood cell parameters (HGB, HCT, MCV), platelet parameters (MPV, PCT, P-LCC), and reticulocyte parameters (RET, LFR, RHE).

### 4.6. Histopathological Analysis

The harvested tissues were fixed in 10% neutral-buffered formalin for 48 h. After fixation, the samples were dehydrated in ascending concentrations of ethanol and subsequently cleared in xylene baths. Paraffin infiltration was then performed at 58 °C for 5 h, using low-melting-point paraffin. Thin sections of 2 µm thickness were obtained from the paraffin blocks using the rotary microtome. Sections were stained with hematoxylin and eosin (H&E) following standard protocols. Histological samples were examined under an Olympus BX51 microscope (Olympus Life and Material Science Europa GMBH, Hamburg, Germany) and the bright field images were obtained with an Olympus SP350 (Olympus Life and Material Science Europa GMBH) digital camera and processed using the Olympus CellSens software (version 2.1; Olympus). Histological scoring was based on the degree of colon inflammation, following the morphological criteria described by Fábrega, M. et al., [71]. (Table 2). Full-thickness distal colon sections were selected and evaluated microscopically, as this region is considered a primary site of inflammation in DSS-induced chronic colitis, allowing a consistent and representative assessment of tissue injury, including the mucosal epithelium, lamina propria, crypt architecture, submucosa, and muscularis layer.

### 4.7. Immunohistochemistry Analysis

For CD3 immunolabelling (clone LN10, Leica Biosystems Newcastle Ltd., ready-to-use), tissue sections were processed automatically using the Leica Bond-Max™ immunohistochemistry system (Leica Biosystems, Melbourne, Australia; Bond Max model M2, series 12,154). Immunoreactivity was identified by the presence of brown cytoplasmic staining pattern. Appropriate positive control tissues were included in each run. Negative controls were performed by substituting the primary antibody with normal serum from the same species as that of the primary antibody.

### 4.8. Statistical Analysis

Data are expressed as mean ± standard deviation (SD), with five animals per group. Statistical analyses were performed using GraphPad Prism 10 (San Diego, CA, USA). Differences between groups were evaluated using one-way analysis of variance (ANOVA), followed by Dunnett’s post hoc test for multiple comparisons. A *p*-value < 0.05 was considered statistically significant.

## 5. Conclusions

This study provides evidence for the protective effects of EECM in a chronic DSS-induced colitis model. The extract improved clinical parameters and reduced intestinal injury associated with repeated DSS exposure. Histological evaluation confirmed preservation of colonic architecture and decreased inflammatory infiltration. Moreover, immunohistochemical analysis showed reduced CD3-positive T-lymphocyte infiltration, suggesting modulation of immune cell infiltration and attenuation of T-lymphocyte-mediated inflammatory activity within the colon. Overall, these findings indicate that EECM contributes to the attenuation of chronic intestinal inflammation while supporting mucosal integrity. Further studies are required to clarify the underlying mechanisms and evaluate their potential application in inflammatory bowel disease.

## Figures and Tables

**Table 1:** Disease Activity Index (DAI) Scores.

Weight Loss%	Stool Traits	Fecal Occult Blood/Gross Blood in the Stool	Scores
0	Normal	Normal	0
1–5	Slightly loose	Slightly bloody	1
5–10	Loose	Bloody	2
10–15	Very loose	Gross bleeding gross blood in the stool	3
>15	Watery diarrhea	Massive bleeding	4

**Table 2:** Microscopic scoring criteria of full-thickness distal colon sections.

Mucosal Epithelium and Lamina Propria	Crypts	Submucosa	Muscularis Layer
-Ulceration: none (0); mild surface (0–25%) (1); moderate (25–50%) (2); severe (50–75%) (3); extensive-full thickness (>75%) (4). -Polymorphonuclear cell infiltrate -Mononuclear cell infiltrate and fibrosis -Edema and dilatation of lacteals -Crypts	-Mitotic activity: lower third (0); mild mid third (1); moderate mid third (2); upper third (3) -Dilatation -Goblet cell depletion	-Polymorphonuclear cell infiltrate -Mononuclear cell infiltrate -Edema	-Polymorphonuclear cell infiltrate -Mononuclear cell infiltrate -Edema -Infiltration in the serosa

In addition to the criteria described above, microscopic changes were graded according to severity using a standard scoring system: 0 = no significant change; 1 = minimal; 2 = mild; 3 = moderate; 4 = severe. For samples containing two transverse sections of the colon an average of the scores of the two sections was calculated.

## Data Availability

Data is contained within the article or Supplementary Materials.

## Supplementary Materials

The following supporting information can be downloaded at: https://www.mdpi.com/article/10.3390/molecules31091401/s1, Table S1: The content of phenolic compounds in ethanolic extracts of C. *micranthum* leaves by HPLC-DAD-ESI-MS (mg/g of extract).
