# Supplementary material for: Therapeutic Potential of Kinkeliba (Combretum micranthum G. Don) Ethanolic Extract in Chronic DSS-Induced Colitis
Source: Molecules. 2026 Apr 23;31(9):1401. doi: 10.3390/molecules31091401 (PMC13165367; doi:10.3390/molecules31091401)
Supplement: Supplementary file 1 [file molecules-31-01401-s001.zip › molecules-4259996-supplementary.pdf]

Article

Therapeutic Potential of Kinkeliba (*Combretum micranthum* G. Don) Ethanolic Extract in Chronic DSS-Induced Colitis

Ibrahima Mamadou SALL[1*], Meriem AZIEZ[2], Dragoş Hodor[1], Alina Diana HAŞAŞ[3] Mara-Georgiana HARALAMBIE[1], Semzenisi Ecaterina[1], Alexia-Teodora Hota[1], and Alexandru-Flaviu TĂBĂRAN [1*]

[1] Department of Anatomic Pathology, Faculty of Veterinary Medicine, University of Agricultural Sciences and Veterinary Medicine of Cluj-Napoca, Romania.
[2] Laboratory of Plant Biotechnology and Ethnobotany, Faculty of Nature and Life Sciences, University of Bejaia, Bejaia 06000, Algeria.
[3] Department of Pathophysiology, Faculty of Veterinary Medicine, University of Agricultural Sciences and Veterinary Medicine of Cluj-Napoca, Romania.

* Correspondence: ibrahima.sall@student.usamvcluj.ro; alexandru.tabaran@usamvcluj.ro

Table S1: The content of phenolic compounds in ethanolic extracts *of C.micranthum* leaves by HPLC-DAD-ESI-MS (mg/g of extract).

	Phenolic Compounds	Subclass	R_t (min)	λmax (nm)	$[M+H]^+$ (m/z)	**Extract** (mg/g)
**1**	Gallic acid	Hydroxybenzoic acid	4.70	275	171	4.80
**2**	Protocatechuic acid	Hydroxybenzoic acid	9.01	280	155	14.14
**3**	1,6-Digalloyl-glucose	Gallotannin	13.16	280	485	5.82
**4**	Ellagic acid-arabinoside	Hydroxybenzoic acid	13.93	270, 360	435	14.04
**5**	Ellagic acid-glucoside	Hydroxybenzoic acid	14.43	270, 360	465	8.08
**6**	Sanguiin H-4	Ellagitannin	15.14	270, 360	635	102.56
**7**	Corilagin	Ellagitannin	16.22	270, 360	635	63.29
**8**	Ellagic acid	Hydroxybenzoic acid	16.63	270, 360	303	12.10
**9**	Combretastatin B1	Stilbene	22.69	275	335	68.71
	**Total phenolics** (mg/g)					**293.54**
